# Epigenetic control of epileptogenesis by miR-146a

**DOI:** 10.18632/oncotarget.18364

**Published:** 2017-06-04

**Authors:** Valentina Iori, Eleonora Aronica, Annamaria Vezzani

**Affiliations:** Department of Neuroscience, IRCCS-Istituto di Ricerche Farmacologiche “Mario Negri”, Milano, Italy

**Keywords:** microRNA, neuroinflammation, epilepsy, seizures, disease-modification

Treatments for epilepsy, a multi-etiology brain disorder affecting over 50 million people worldwide, are so far limited to drugs which provide mere symptomatic control of seizures, have many adverse effects and are ineffective in up to 40% of patients [1]. These considerations urge on the development of therapies with disease-modifying properties targeting the key pathogenic mechanisms generating spontaneous seizures. Epigenetic mechanisms have been recently recognized as important determinants of the pathologic brain modifications occurring in animal models of epilepsy and in human epilepsy-associated pathologies [2]. In particular, modifications in brain levels of various microRNAs (miRNAs) have been described in epilepsy [3]. These small non-coding RNAs, in concert with gene transcriptional changes, control the expression levels of multiple proteins by decreasing mRNA stability and/or translation. The miRNAs brain modifications discovered in epilepsy helped to identify novel mechanisms -or reinforced the pathogenic role of established mechanisms- contributing to neural, glial and vascular network alterations underlying epileptogenesis (the development and extension of brain tissue capable of generating spontaneous seizures). The main experimental approaches used so far to study miRNAs in epilepsy were based on unbiased microarray analysis of epileptogenic brain tissue from animal models at different stages of disease development. This approach allowed to find miRNAs that were differentially expressed compared to control animals [3]. Special attention was paid to miRNAs commonly changed across different experimental models, and validated in human epilepsy tissue obtained from therapeutic surgery. Interventions aimed at either silencing or mimicking the selected miRNA using its synthetic antagomir or oligonucleotide mimic, respectively, were used to prove miRNAs involvement in seizure generation or in the associated neuropathology. This strategy, for example, allowed to link the altered miRNAs in seizure-susceptible brain regions with changes in neuronal dendritic spines, receptor and ion channels regulating inhibitory and excitatory neurotransmission, transcriptional factors, and modulators of the inflammatory response [3]. In this respect, miRNAs targeting inflammatory signals attracted special attention since neuroinflammation has been increasingly recognized as a pathologic hallmark of human pharmacoresistant epilepsies. It consists of the activation of specific innate immune signals in glia and neurons, also involving brain microvasculature. In epilepsy animal models, neuroinflammation arises in response to either brain injury or neuronal activity (neurogenic neuroinflammation) [4]. Animal models showed that the activation of specific innate immunity mechanisms, such as the IL-1 receptor (IL-1R1)/Toll-like receptor (TLR4) pathway, plays an active role in seizure pathogenesis [4]. The IL-1R1/TLR4 axis and its endogenous ligands IL-1β and HMGB1, respectively, were validated as major pathogenic factors in epilepsy based on their induction in experimental and human epilepsy brain, and by showing that their pharmacological or genetic inactivation dramatically reduces experimental seizures [4]. Case report studies in patients with intractable seizures, and a proof-of-concept clinical trial, support the clinical significance of animal findings. The mechanisms underlying the ictogenic effects evoked by IL-1R1/TLR4 axis activation are at least twofold: 1. activation in neurons promotes excito-toxicity and enhances neuronal excitability by enhancing calcium influx via N-methyl-D-aspartate receptors and reduces inhibitory GABA currents. These fast-onset effects are mediated by protein kinases activation [5]; 2. activation in glial cells induces cytokines, chemokines, COX-2 and complement factors by transcriptional activation of NF-κB and AP-1 sensitive genes, thereby perpetuating the neuroinflammatory response. Several of these mediators have neuromodulatory functions which may explain their contribution to network hyper-excitability underlying seizures [5].

The IL-1R1/TLR4 activation after an epileptogenic event may potentially induce a decrease in seizure threshold therefore contributing to establishing a chronic epileptogenic network. If proven to be true, then this signaling represents a potential target for novel drugs which prevent the disease development or reduce its severity and progression.

**Figure 1:**
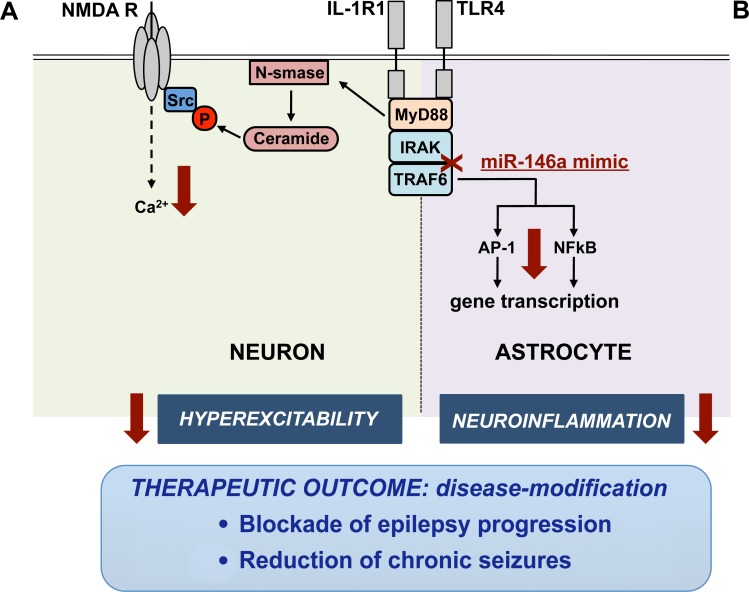
IL-1R1/TLR4 signaling inhibition by miR-146a mimic in neurons and astrocytes The activation of IL-1R1/TLR4 axis promotes seizure generation in susceptible brain areas by a twofold mechanism mediated by IL-1β and HMGB1 activation of neuronal or astrocyte receptors. A. Activation of IL-1R1/TLR4 axis in neurons provokes hyper-excitability by enhancing Ca2+ influx via the glutamate-sensitive N-methyl-D-aspartate receptor (NMDAR). This fast-onset posttranslational response involves ceramide-induced Src kinase phosphorylation (P) of the NR2B subunit of the NMDAR which regulates Ca2+ influx [5]. B. Activation of IL-1R1/TLR4 axis in astrocytes induces NF-κB- and AP-1 dependent transcription of inflammatory genes. miR-146a mimic (red cross) down-regulates IRAK1/2 and TRAF6 intracellular proteins (downward arrow) which are required for IL-1R1/TLR4 signal transduction. IL-1R1/TLR4 axis inhibition by miR-146a mimic -or using a combination of drugs, namely VX-765 that blocks IL-1β biosynthesis, and Cyanobacterial LPS, a TLR4 antagonist [6]- reduces hyper-excitability and neuroinflammation (downward arrow) in mice developing epilepsy, thus resulting in therapeutic outcomes.

A recent study [6] has published discoveries about the therapeutic success of an epigenetic intervention in models of seizures and epilepsy based on the brain delivery of a synthetic mimic of miR-146a which inhibits the IL-1R1/TLR4 intracellular signaling cascade [7]. The study identified that this epigenetic intervention inhibits IL-1R1/TLR4 signaling activation in neurons, thus reducing network excitability in the hippocampus, a key area contributing to seizure generation in various acquired epilepsies. Notably, intracerebroventricular delivery of a miR-146a mimic in a mouse model of epileptogenesis for only 2 weeks after the onset of epilepsy blocked the progression of the disease thus reducing dramatically the frequency of chronic spontaneous seizures. This study provides the first proof-of-concept evidence for disease-modification using an intervention transiently applied after epilepsy onset. Differently, carbamazepine a clinically used antiepileptic drug, did not prevent disease progression and its inhibitory effect on seizures rapidly elapsed after drug discontinuation. The therapeutic effects of the synthetic mimic were associated with increased miR-146a levels in neuronal cells and astrocytes in forebrain regions involved in the epileptic circuitry. Increased miR-146a in human astrocytes was shown to blunt IL-1R1/TLR4 axis mediated release of inflammatory cytokines [8]. It is therefore conceivable that the mechanisms underlying the miR-146a mimic's therapeutic effects relay upon inhibition of the IL-1R1/TLR4 pathway in both neurons and glia (Figure 1). Transcriptomic analysis of differentially expressed genes in the hippocampus of mice injected with miR-146a mimic vs control mice will help to unveil the molecules and pathways most significantly affected by this therapeutic intervention. In fact, a better understanding of the epigenetic regulation of pathophysiological mechanisms in epilepsy may lead to the discovery of novel drug targets. In support, the miR-146a mimic's therapeutic effects were fully reproduced in the murine epilepsy model by a combination of investigational drugs blocking the IL-1R1/TLR4 axis. The transient targeting of this immune-related signal in human epilepsies with unfavorable prognosis may provide an innovative treatment approach for improving the disease course by acting on a pathogenic mechanism. This provides an added clinical value as compared to chronic administration of the clinically used drugs that afford a mere symptomatic control of seizures.
